# Screening Candidate Genes at the *Co* Locus Conferring to the Columnar Growth Habit in Apple (*Malus × Domestica* Borkh.)

**DOI:** 10.3390/genes14050964

**Published:** 2023-04-24

**Authors:** Jing Guo, Yuan Zhao, Yu Chu, Yuru Li, Yuqi Song, Qi Pan, Zhannan Qiu, Boyang Yu, Yuandi Zhu

**Affiliations:** Department of Pomology, College of Horticulture, China Agricultural University, Yuanmingyuan West Road No. 2, Haidian District, Beijing 100193, China

**Keywords:** *Malus × domestica* Borkh., columnar apples, candidate gene screening, subcellular localization, ectopic expression

## Abstract

The columnar growth trait of apple (*Malus* × *domestica* Borkh.) is genetically controlled by the Columnar (*Co*) locus on 10 chromosomes, including several candidate genes. Except for *MdCo31*, other candidate genes at the *Co* locus are less elucidated. In this study, a strategy of step-by-step screening was adopted to select 11 candidate genes by experimental cloning, transient expression, and genetic transformation. There existed several SNPs in four genes by sequence alignment in columnar and non-columnar apples. Two genes were detected in the nucleus and three genes in the cell membrane, other genes were located in multiple cellular structures by subcellular location. Ectopic expression demonstrated that more branching occurred in *MdCo38*-OE by upregulating *NtPIN1* and *NtGA2ox* and enlarged leaves in *MdCo41*-OE tobaccos by upregulating *NtCCDs*. Transcripts of *MdCo38* and *MdCo41* were associated with the *Co* genotypes in apples. The results indicate that *MdCo38* and *MdCo41* are involved in the columnar growth phenotype in apple, probably through altering polar auxin transport, active gibberellin levels, and strigolactone biosynthesis.

## 1. Introduction

Tree architecture is a critical agronomic trait of fruit trees, directly influencing planting density and canopy, and subsequently yield and quality of fruits [1,2]. Columnar apples are characterized by compact growth with less branching and more spurs in the apple (*Malus × domestica* Borkh.) and are genetically controlled by the *Co* locus at chromosome 10 in the apple genome [3,4]. The columnar tree form is promising for future orchard production due to its labor-saving characteristics and mechanical management [5]. Therefore, identification of the nature of the *Co* gene is an effective way to realize gene-targeted apple breeding via genetic transformation. 

According to the tree growth habit and their fruit-bearing, apple trees have been divided into four ideotypes: columnar, standard, spur, and tip bearer [6]. The columnar apple is presented by ‘Wijcik McIntosh’ (or ‘Wijcik’), a bud sport from the standard apple cultivar ‘McIntosh’ [5]. Due to the compact growth, short internodes, and few lateral branches, this growth habit is referred to as ‘columnar’ and is controlled by a single dominant *Co* (columnar) [7,8]. The first linkage analysis of the *Co* gene is conducted by RAPD (random amplified polymorphic DNA) markers using a segregation population crossed by ‘Rome Beauty’ × ‘White Angel’, mapping the *Co* at the linkage group 10 [9]. An SSR (Simple Sequence Repeats) maker, SSRco165, is developed from F_1_ populations of ‘Wijcik’ × ‘NY75411-67’ and ‘Wijcik’ × ‘NY75411-58’, linked to *Co* at 2.9 cM (centiMorgans) [10]. WB82_670_, an SSR marker obtained from the progeny of ‘Fuji’ × ‘Tuscan’, is closely linked to *Co* at 1.8 cM [11]. The genetic distance of a SCAR (Sequence Characterized Amplified Regions) marker, SCAR_682_, derived from the cross of ‘Fuji spur’×‘Telamon’, is 2.9 cM to *Co* [12]. Another SSR marker, CH03d11, is linked to *Co* at 3.9 cM and developed from ‘Fuji’ × 8H-9-45 and ‘Fuji’ × 5-12786 [13]. The abovementioned DNA molecular markers are widely applied for the genetic mapping of *Co*.

Several physical maps of *Co* have been reported since the reference genome of ‘Golden Delicious’ was published [14], such as Bai et al. [15], Baldi et al. [16], Moriya et al. [17], Wolters et al. [18], and Otto et al. [19]. Based on the BAC (bacterial artificial chromosome) library sequencing of several apple varieties and a large number of apple hybrid populations, the *Co* is finely mapped at the region of 18.51~19.1 Mb on chromosome 10 [15,16,17,18,19]. An 8.2kb retrotransposon, Ty3/Gypsy-44 (referred to as Gy-44), is inserted into the 5′ LTR of another retrotransposon Ty/Gyspy-33 (Gy-33) in the ‘Wijcik’ genome, forming a nested retrotransposon associated with the columnar mutation [19]. Among many predicted genes, *MdCo31* (MDP0000687812) encoding 2OG-Fe(II) oxygenases (2-oxoglutarate and Fe(II) dependent oxygenases) is a strong candidate gene for *Co* [18]. *MdCo31*, the same as *dmr6-like* [20], *MdDOX-Co* [21], and *MdCoL* [22], is highly expressed in the shoot tips of columnar apples, and is negatively correlated with dwarf growth and shortened internode in apples by preventing 12-hydroxylation of GA12 biosynthesis to reduce active GAs in planta [22,23,24]. Other predicted genes at the *Co* locus are less deciphered. We suspect that other candidate genes of *Co* are also involved in columnar growth in apples.

In our previous study, 15 candidate genes covering the *Co* region (18.51~19.1 Mb) are selected by SRA/EST (Sequence Read Archive/Expressed Sequence Tags) database mining and differential expression in shoot tips of ‘Wijcik’ and ‘McIntosh’ by qRT-PCR analysis. In this study, candidate genes were identified by genomic DNA and cDNA sequencing and sequence alignments between ‘Wijcik’ and ‘McIntosh’ apples. Subcellular localization and tobacco (*Nicotiana benthamiana* L.) transformation were conducted to analyze the biological functions of these genes. Those candidate genes that led to morphological changes in transgenic plants were verified by gene expression analysis in F1 populations crossed by columnar apples. Screening candidate genes at the *Co* locus helps to elucidate molecular mechanisms of the columnar mutation and serves as a directional genetic improvement for apple trees.

## 2. Materials and Methods

### 2.1. Plant Materials

The columnar apple cultivar ‘Wijicik’, non-columnar apple ‘McIntosh’, and F1 hybrid population of ‘Jinlei No.1’ × ‘Maypole’ cultivated at the Shangzhuang experimental station of China Agricultural Universtity (40°08′ N, 116°11′ E) were used for amplified *Co* candidate sequences, qRT-PCR, and *Co* genotype [25]. Tobacco seeds were surface sterilized using 70% ethanol for 30 s and 2% NaClO for 10 min and geminated on MS (Murashige and Skoog) medium plus 30 g/L sucrose, and were used for genetic transformation after the growth of 4–5 leaves. The materials required for qRT-PCR were collected from tobacco leaves and tips 30 days after transplanting in the greenhouse, and the samples were frozen in liquid nitrogen and stored in a −80 °C refrigerator.

### 2.2. Extraction of DNA and RNA

Total DNA was extracted using a modified CTAB method according to Doyle [26]. DNA purity and concentration were measured using a NanoPhotometer^®^ spectrophotometer (Implen, Westlake Village, CA, USA).

Total RNA was extracted using FastPure^®^ Plant Total RNA Isolation Kit (polysaccharides and polyphenolics-rich) (Vazyme, Nanjing, China). The cDNA was obtained by using the PrimeScriptTM 1st Strand cDNA Synthesis Kit (6110A, TaKaRa, Dalian, China).

### 2.3. DNA Sequencing and Bioinformatic Analysis

The apple ‘Golden Delicious’ genome (accessed via https://www.rosaceae.org/Analysis/10816131, accessed on 5 July 2022) was used for searching DNA sequences. Twelve specific primer pairs of candidate genes (Table S1) were designed for PCR amplification. Both genomic DNA and cDNA of apple were used as templates. The following PCR parameters were set at 95 °C for 5 min, followed by 40 cycles at 95 °C for 5 s, 56–58 °C for 30 s, and 72 °C for 2 min. The annealing temperature was adjusted according to the primer itself. The PCR products were separated by 1% agarose gel electrophoresis. Sequences of gDNA and cDNA in both ‘McIntosh’ and ‘Wijcik’ were used for alignment analysis by DNAMAN. Phylogenetic analysis was performed by MEGA11.0. The subcellular localization of *Co* candidate genes was predicted using Plant-mPLoc (accessed via http://www.csbio.sjtu.edu.cn/bioinf/plant-multi/, accessed on 2 January 2023).

### 2.4. Subcellular Localization of Candidate Genes

The full-length CDS (coding sequence) of *MdCo41* was amplified with the forward primer containing an *Xma*I restriction site and the reverse primer containing *Hind*Ⅲ restriction sites. Forward primers with an *Xma*I and reverse primers with an *Xba*I restriction site were designed to amplify other candidate genes (Table S1). The amplification products without the terminator codon of each candidate gene were ligated to the pSuper1300-GFP vector for constructing the translational GFP fusion constructs. The recombinant plasmid was transferred into *Agrobacterium tumefaciens* strain GV3101, and incubated at 28 °C, 200 rpm. Bacterial solution was collected when OD600 was at 0.8–1.0, and resuspended using 10 mM MES, 10 mM MgCl_2,_ and 100 mM AS to injection. Tobacco seedlings from two-week-old infected tobaccos were incubated for 1 d in darkness and 2 d in light, and observed by confocal laser scanning microscope (OLYMPUS FLUOVIEW FV3000, Japan Olympus Corporation, Tokyo, Japan).

### 2.5. Transformation of Tobacco

Tobacco seeds were surface sterilized according to the previous protocols. The CDS of each candidate gene was ligated into a pBI121 vector, and fusion vectors were transformed into tobacco by *A*. *tumefaciens* EHA105 and incubated at 28 °C, 200 rpm. Bacterial solution was collected when OD600 was at 0.5–0.8, and resuspended using MS medium plus 30 g/L sucrose and 100 mM AS. Transgenic tobacco discs grown in MS medium were supplemented with 30g/L sucrose, 2 mg/L 6-BA, and 0.2 mg/L IAA for 3d then transferred to MS plus 2 mg/L 6-BA, 0.2 mg/L IAA, 100 mg/L kanamycin, and 250 mg/L cefotaxime, and changed for 15d until regeneration shoots growth.

### 2.6. Quantitative Real-Time PCR (qRT-PCR)

Ectopic transgenic tobaccos in greenhouse and F1 populations of ‘Jinlei No.1’ × ‘Maypole’ shoots were collected. The qRT-PCR assay was performed using the SuperReal.

PreMix Plus (SYBR Green) (Tiangen, Beijing, China). The qRT-PCR program was as follows: 94 °C for 30 s and then 40 cycles of 95 °C for 5 s and 60 °C for 30 s. The primers used for qRT-PCR were, as seen in Table S1. The relative expression levels in each line were normalized against the expression of the internal standard, *NtACTIN*, and *MdACTIN*, respectively. The 2^−ΔΔCt^ method was used to calculate relative gene expression levels [27].

### 2.7. Statistical Analysis

The plant height, number of branches, leaves area, and internode length of transgenic tobacco were recorded 60 days after the tobacco was transplanted into a greenhouse. Additionally, using SPSS17.0 to analyze the statistical data, differences of *p* < 0.05 and *p* < 0.01 were considered significant and extremely significant levels, respectively. Graphs were prepared using GraphPad Prism 9. All of the samples were analyzed in three replicates.

## 3. Results

### 3.1. Identification and Sequence Alignments of Co Candidate Genes

**Figure 1:**
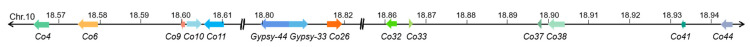
Schematic locations of *Co* candidate genes and the retrotransposon insertion on chromosome 10 in ‘Wijcik’ apple.

In order to confirm the genomic DNA sequence difference between ‘Wijcik’ (columnar) and ‘McIntosh’ (non-columnar), genomic DNA and cDNA were used to identify candidate genes. Among 15 candidate genes, 12 were isolated from two apple cultivars, including *MdCo31* (Figure 1, Table S2), whereas no PCR amplification was obtained for *Co27, Co28*, and *Co30* from both gDNA and cDNA templates. The reasonable explanation was pseudogenes annotated in the apple reference genome. Twelve candidate genes were mapped on chromosome 10 based on their physical positions, and the retrotransposon insertion was in between *Co11* and *MdCo31* (Figure 1).

**Figure 2:**
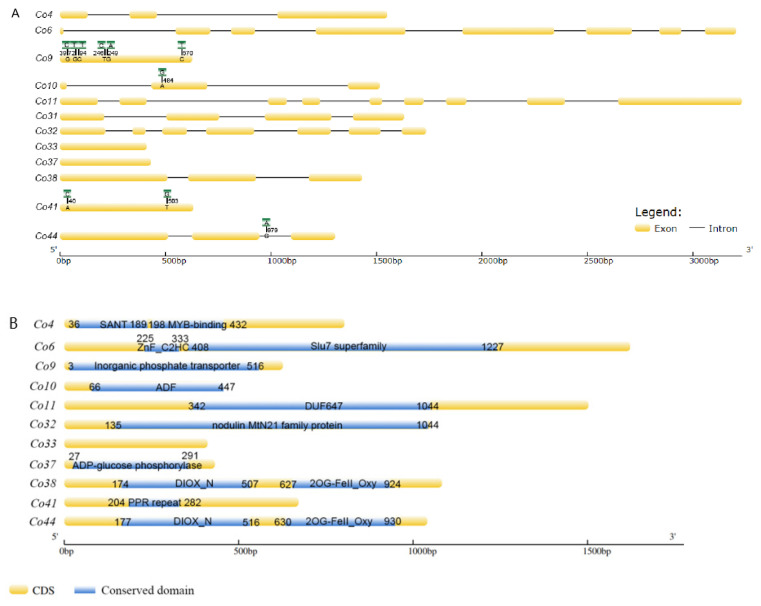
Schematic structures (**A**) and conserved domains (**B**) (blue marked) of candidate genes between ‘Wijcik’ (green marked SNPs) and ‘McIntosh’ (yellow marked) apples.

*Co9*, a putative inorganic phosphate transporter, exhibited six nucleotide variations in CDS by sequence alignments, including two nucleotide transversions and four nucleotide transitions in ‘Wijcik’, compared to ‘McIntosh’ apple (Figure 2A). Only one nucleotide at the 94th bp transit from C to T, leading to a deduced amino acid, Leu to Phe, a non-conserved amino acid, even though this transition was present in the conserved domain (Figure 2B). *Co10* encoded a putative actin depolymerization factor (ADF), in which one nucleotide transition (A to G) at the 484th bp of CDS did not change its amino acid sequence (Figure 2). *Co41*, containing 5 pentatricopetide repeat (PPR) motifs, had two transversions at the 40th (A to C) and 503rd bps (T to G) in the non-conserved domain of the ‘Wijcik’ DNA sequence (Figure 2). *Co44* encoded a putative *ACO1*-like gene that contained DIOX-N (non-haem dioxygenase in the morphine synthesis N-terminal) and 2OG-Fe (Ⅱ) oxygenase domains, in which one nucleotide transverses in the intron of genomic DNA in ‘Wijcik’ and ‘McIntosh’ (Figure 2). No sequence difference was found in seven other genes. 

*Co4* encoded a putative MYB transcription factor, containing a SANT (switching-defective protein 3, adaptor 2, nuclear receptor corepressor, and transcription factor IIIB) DNA binding domain and MYB-binding domains. There were Slu7 (pre-mRNA splicing factor) and zinc figure domains in *Co6*, and the ADF (actin depolymerization factor) domain in *Co11*. *Co32* encoded putative walls are thin 1 (WAT1)-related proteins, including the nodulin MtN21 domain. *Co37* functioned probably as ADP-glucose phosphorylase. *Co38* comprised DIOX-N and 2OG-Fe (Ⅱ) oxygenase domains, similar to *Co44*, a homolog with the *ACO1*-*like* gene. No conserved domain was predicted in *Co33*, and its function is unknown (Figure 2B). 

### 3.2. Subcellular Localization Analysis of Co Candidate Genes in Tobacco

**Figure 3:**
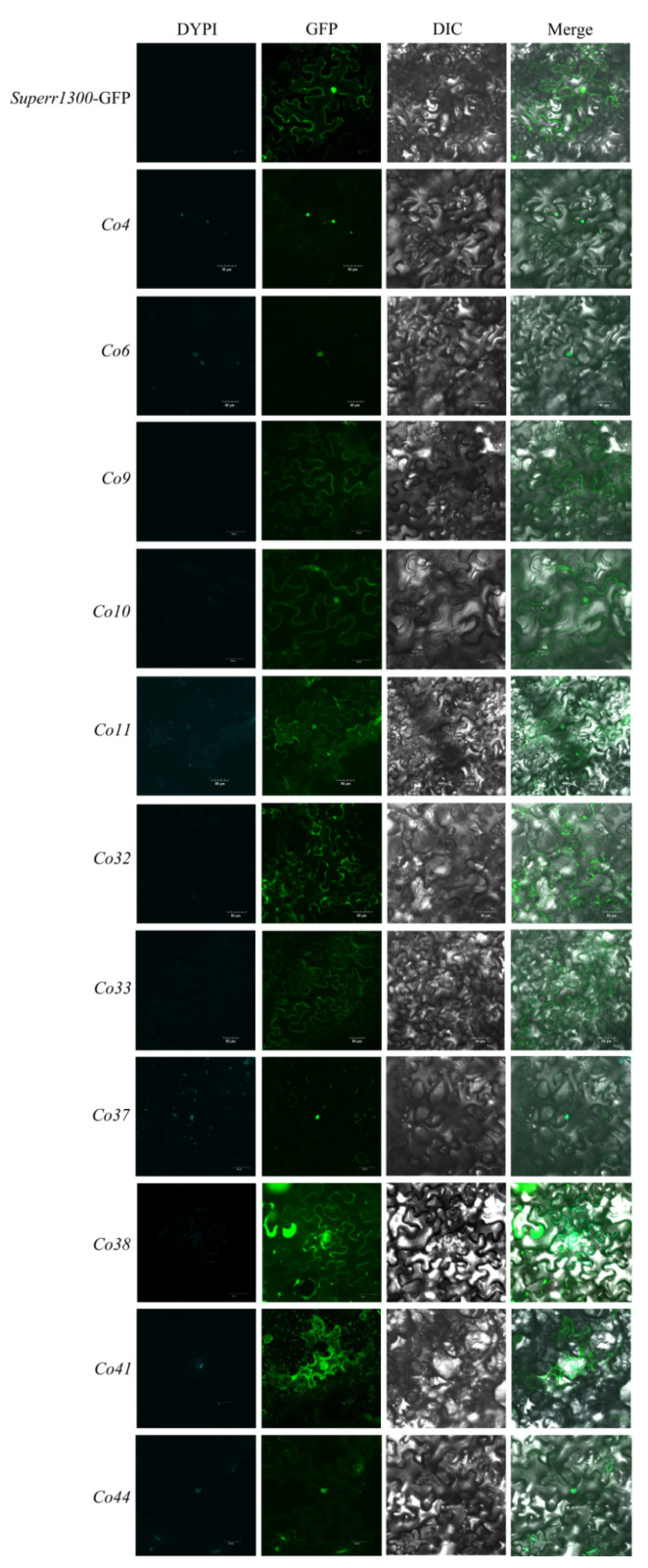
Subcellular localization of *Co* candidate genes.

To determine the subcellular localization of candidate genes, fusion proteins of Co-GFP were constructed and transiently expressed in *N. benthamiana* leaves using *A. tumefaciens* infiltration. Protein localization was examined 48 h after infiltration and representative images are shown in Figure 3. Co4- and Co6-GFP fusion proteins were detected in the nucleus; Co9-, Co33-, and Co38-GFP in cell membranes; Co10-GFP in the cell membrane and nucleus; Co32-GFP in cell membranes and cytoplasm; Co11- and Co41-GFP in the cytoplasm, cell membrane, and nucleus; Co37- and Co44-GFP in the nucleus and cytoplasm (Figure 3). Cellular locations of *Co4*, *Co6*, *Co9*, *Co11*, *Co32*, *Co37*, *Co41*, and *Co44* were consistent with silico prediction, whereas contradictory prediction occurred for *Co10* and *Co38* (in the cytoplasm), and *Co33* (in the nucleus).

### 3.3. Ectopic Expression Analysis of Co Candidate Genes in Tobacco

**Figure 4:**
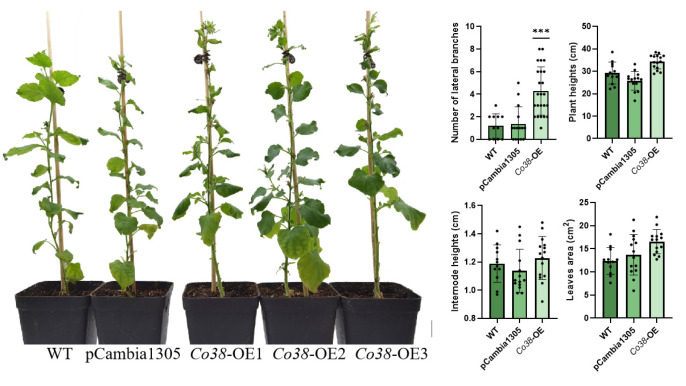
Phenotypes of transgenic tobacco overexpressed by *MdCo38.* Bar, 1 cm. *** represents a significant difference of *p* < 0.0001.

**Figure 5:**
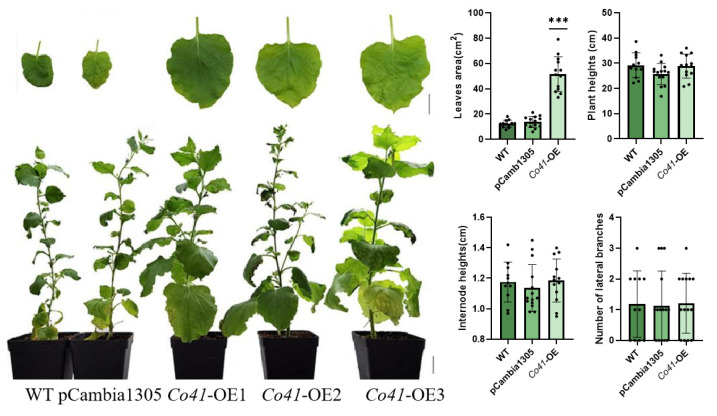
Phenotypes of transgenic tobacco overexpressed by *MdCo41.* Bar, 1 cm. *** represents a significant difference of *p* < 0.0001.

To verify the biological function of candidate genes, four genes (*Co32*, *Co33*, *Co38*, and *Co41*) upregulated in the columnar apple ‘Wijcik’ were selected for ectopic expression in *N. benthamiana*. Morphological alterations were observed in *Co38*-OE and *Co41*-OE tobaccos (Figure 4 and Figure 5), but no significant difference in *Co32*-OE and *Co33*-OE plants was observed compared with wild-type tobacco (WT) and transformants of the pCambia1305 vector (EV) (Figure S1). 

Overexpression of *MdCo38* resulted in increased branching in T_0_ tobaccos. *MdCo38*-OE plants had branching numbers with an average of 4.29 (±2.14), significantly more than those in WT 1.18 (±1.07) and EV 1.33 (±1.54) plants (Figure 4). No significant changes were observed in transgenic tobaccos for other morphological traits, such as plant height (34.29 cm ± 3.21), internode length (1.23 cm ± 0.15), and leaf size (17.19 cm^2^ ± 3.52) compared to controls (Figure 4). Overexpression of *MdCo41* led to leaf enlargement (51.58 cm^2^ ± 13.77) compared to that of WT (12.37 cm^2^ ± 2.89) and empty vector (13.71 cm^2^ ± 4.37) plants (Figure 5). No obvious alteration in plant height, internode length (1.19 cm ± 0.14), and number of lateral branches (1.21 ± 0.97) occurred in *MdCo41*-OE plants compared to controls (Figure 5).

### 3.4. Branching Was Stimulated by High Expression of NtPIN1 and NtGA2ox in MdCo38-OE Plants

**Figure 6:**
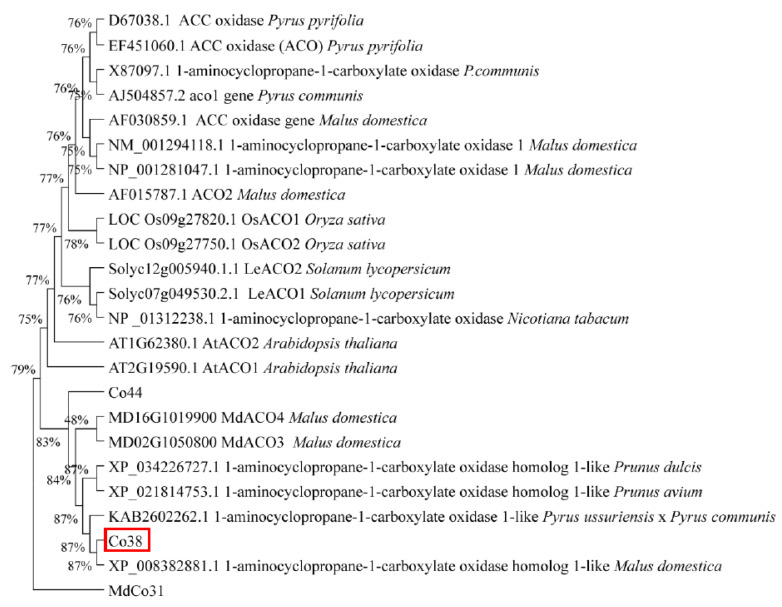
Phylogenetic analysis of *MdCo38* with its homologs in plants.

**Figure 7:**
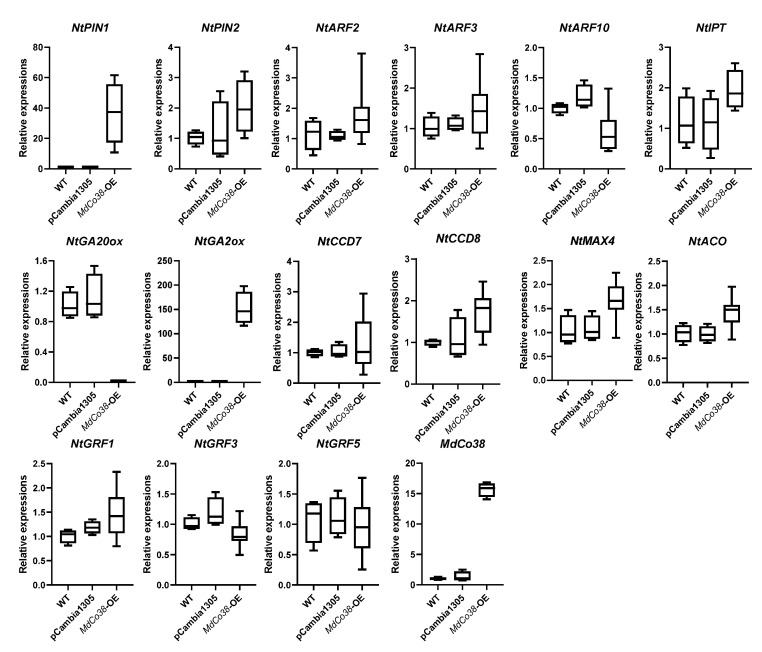
Expression analysis of several genes related to hormone metabolism and growth regulation factors in *MdCo38*-OE plant.

Phylogenetic analysis showed that *MdCo38* was clustered with a few *ACO-like* genes in plants, suggesting a similar function as the *ACO* (Figure 6). Several key genes related to auxin, gibberellin (GA), and strigolactones (SLs) metabolism were analyzed by qRT-PCR. The auxin efflux carrier gene, *NtPIN1*, and *NtGA2ox* were significantly upregulated, but *NtGA20ox* was downregulated in *MdCo38*-OE plants. No significant differential expression was detected in transgenic plants, including *NtPIN2*, *NtARF2* (auxin response factor), *NtARF3*, *NtARF10*, *NtCCD7*, *NtCCD8*, *NtACO*, *NtMAX4*, the isopentenyl transferase gene *NtIPT*, *NtGRF1* (growth-regulating factor), *NtGRF3*, and *NtGRF5* (Figure 7). The results indicated that the branching of *MdCo38*-OE plants was mainly affected by auxin polar transport and active GA levels.

### 3.5. Leaf Was Enlarged by High Expression of NtCCDs in MdCo41—OE Plants

**Figure 8:**
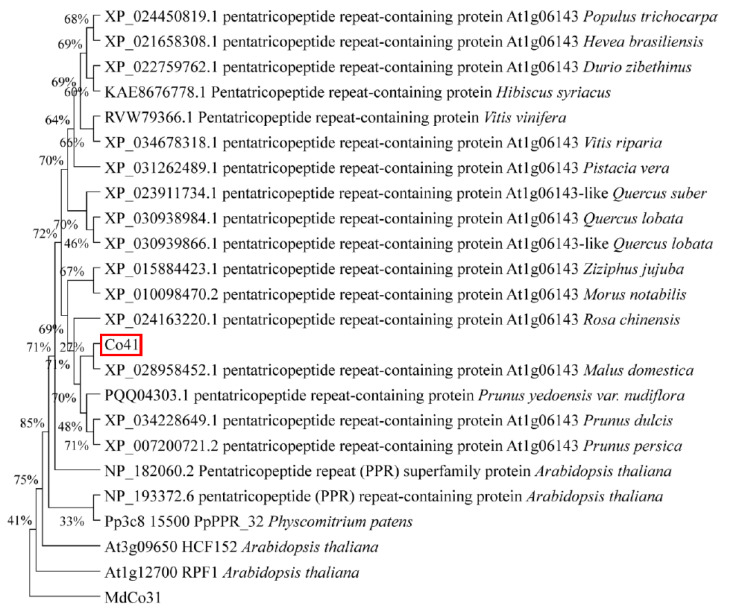
Phylogenetic analysis of *MdCo41* with its homologs in other plants.

**Figure 9:**
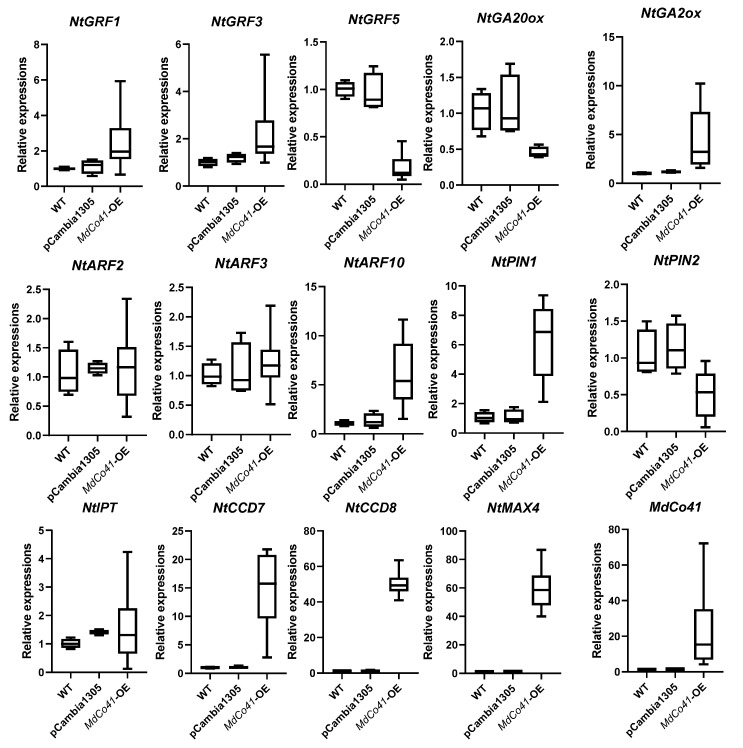
Expression analysis of several genes related to hormone metabolism and growth regulation factors in *MdCo41*-OE plants.

Phylogenetic analysis showed the *MdCo41* homolog with a predicted PPR (XP_028958452.1) in *Malus* (Figure 8). Analysis of qRT-PCR showed that high expression levels of *NtCCD7*, *NtCCD8*, *NtMAX4*, *NtPIN1,* and *NtARF10* were positively correlated with the expression of *MdCo41* (Figure 9). No significant differential expression was observed in transgenic tobaccos, including *NtPIN2*, *NtARF2*, *NtARF3*, *NtGA2ox*, *NtGA20ox*, *NtIPT*, *NtGRF1*, *NtGRF3*, and *NtGRF5* (Figure 9). The results suggested that the overexpression of *MdCo41* altered leaf size by probably regulating the expression of SLs-related genes.

### 3.6. Expression of MdCo38 and MdCo41 in Apple Cultivars and F1 Generation

**Figure 10:**
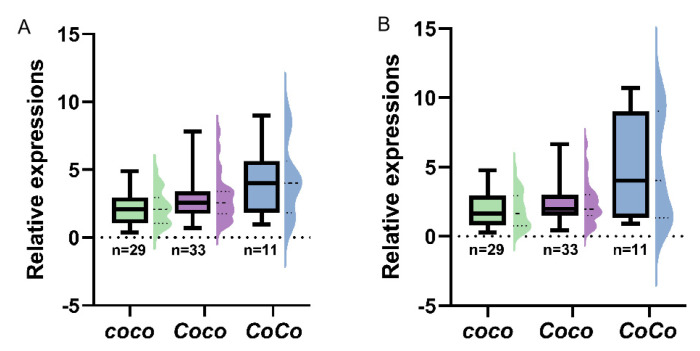
Expression analysis of *MdCo38* (**A**) and *MdCo41* (**B**) in the F_1_ population crossed by columnar apples.

In order to analyze transcriptions of *MdCo38* and *MdCo41* in apples, 68 seedlings were sampled from an F1 population of columnar apples. The genotypes of *Co* were determined by the retrotransposon specific primers in three, *CoCo*, *Coco,* and *coco*. The expression levels of *MdCo38* were 2-fold higher in the *CoCo* groups, than those in groups of *Coco* and *coco* (Figure 10A, Figure S3A). The similar gene expression pattern was found for *MdCo41* (Figure 10B, Figure S3B). It proved that *MdCo38* and *MdCo41* were involved in columnar growth in apple.

## 4. Discussion

Eleven candidate genes at the *Co* locus were analyzed by experimental cloning, bioinformatics analysis, and subcellular location analysis. Among these genes, functions of four upregulated expression genes in ‘Wijcik’ apple were characterized by ectopic overexpression of tobacco. Two candidate genes, *MdCo38* and *MdCo41*, led to phenotypic changes in transgenic plants in terms of plant growth.

### 4.1. Predicted Functions of Genes Downregulated in ‘Wijcik’ Apple

In this study, 11 genes were identified by differential expression analysis between ‘Wijcik’ and ‘McIntosh’ apple cultivars, among which *Co4*, *Co6*, *Co9*, *Co10*, *Co11*, *Co37*, and *Co44* were downregulated in ‘Wijcik’ apple. *Co4*, a member of the MYB transcription factor family, shared high sequence similarity with *MYB30* involved in photomorphogenesis and systemic reactive oxygen species (ROS) signaling that is responsive to light stress in Arabidopsis [28,29]. *Co6* was predicted as a pre-mRNA-Splicing factor, SLU7, that recognizes the A and G 3′ terminals during the second catalytic step in the pre-mRNA splicing process [30,31]. *Co9* shared high homology with other inorganic phosphate transporter proteins 1–4 in plants (Figure S2). *AtPht1-4* in Arabidopsis and *OsPht1-4* (*OsPT4*) in rice enhance the accumulation of inorganic phosphate preferentially in roots in response to phosphorus starvation in transgenic plants [32,33]. Homologs of *Co10* encode ADF family genes, which involve in plant growth, development, and stress response. *MdADF1* transcription accumulates in pollen to promote pollen germination [34]. *AtADF6* expresses in the vascular tissue of all organs [35]. *OsADF11* persistently expresses in different growth stages of rice, mainly in the stem and leaf blade, and responds to salt and drought [36]. The homolog of *Co11* is *RUS2* in Arabidopsis, which contains a DUF647 domain and is involved in early seedling morphogenesis and development by maintaining polar auxin transport [37]. *Co37* encoded a putative ADP-glucose phosphorylase, localized in the nucleus and cytoplasm (Figure 2B and Figure 3). Its homolog, At5g18200.1, catalyzes adenylyl transfer in the reaction of ADP-glucose with various phosphates [38]. *Co44* displayed similar conserved domains but low sequence similarity to *Co38* (Figure 6), probably functioning as ACC oxidase. Further experiments are necessary to explain the roles of these genes in apple.

### 4.2. Functional Analysis of Upregulated Genes in ‘Wijcik’ Apple Co32 and Co33

Heterologous overexpressions of *Co32* and *Co33* exhibited no morphological changes in transgenic tobaccos, although two genes were upregulated in ‘Wijcik’ apple shoot tips (Figure S1, Table 1). *Co32* encoded a plant-specific WAT1-related protein. *AtWAT1* in Arabidopsis, a homolog to *MtN21* (*Medicago truncatula NODULIN21*), is essential for auxin homeostasis and secondary cell wall formation. *AtWAT1* preferentially accumulates in vascular tissues, including in xylem vascular and fiber development. The loss function of *AtWAT1* prevents cell elongation and the formation of secondary cell walls in xylem fibers, which could be restored by applying auxins [39]. Overexpression of *Co32* did not change the phenotypes in transgenic tobaccos compared to CK and EV (Figure S1), regarding plant height, internode length, leaf size, and branching. According to previous studies, columnar apples have thicker branches than non-columnar apples [40], indicating *Co32*’s possible function in cell wall development. To date, the function of *Co33* is not reported.

*MdCo38* and *MdCo41* were highly expressed in columnar apples. Homologs of *MdCo38* encode ACO1, the rate-limiting enzyme in ethylene biosynthesis. Overexpression *PttACO1* in poplar (*Populus tremula* × *tremuloides*) stimulates cambial cell division and inhibits height growth due to elevated endogenous ethylene production [41]. *ACO1*-deficient mutants in rice (*Oryza sativa*) exert shorter internodes than WT. Oppositely, the gain-of-function of *ACO1* gives rise to elongated internodes [42]. *Co41* was grouped in P-class based on the conserved PPR domains [43]. Most of the P-class PPRs are targeted to either mitochondria or plastids, which function principally in RNA processing in plants [43]. A mitochondria P-class gene, *PRECOCIOUS1 (POCO1)*, in Arabidopsis affects mitochondrial RNA editing and functions during flowering time and in the abscisic acid signaling pathway [44]. Arabidopsis *pTAC2*, targeted to plastids, is required for chlorophyll accumulation; consequently, the *ptac2* mutant demonstrates an etiolated appearance and slower growth than the wild type [45]. *MdCo41* was distributed in the cell nucleus, cytoplasm, and cell membranes, indicating an analogous function in mediating gene expression in organelles.

### 4.3. MdCo38 and MdC41 Involved in Columnar Growth in Apple

Plant growth is regulated by a complex network between hormones, especially auxins, cytokinin (CK), GA, and SLs. Several hormone-related genes were selected to analyze the relationship between the phenotype of transgenic plants and gene expression. In tobacco, *PIN1/2* is involved in polar auxin transport, and *ARF2/3/10* in the auxin signaling pathway. *AtPIN1* in *Arabidopsis* mediates organogenesis and vascular tissue differentiation [46], while *AtPIN2* responds to root gravitropic growth by impairing basipetal auxin transport [47]. *OsPIN1* deficiency promotes the tillering and elongation of rice by reducing auxin transport [48]. The loss function of *AtARF2/3/4* leads to smaller leaves by binding promoters of *WOX1* (WUSCHEL-RELATED HOMEOBOX 1) and *PRS* (PRESSED FLOWER), thus suppressing their expressions in Arabidopsis [49]. Overexpression *NtARF10* enlarges the leaf size of transgenic Arabidopsis by increasing cell number and cell size [50]. Our results showed that *NtPIN1* was strongly expressed in *MdCo38*-OE plants, while *NtPIN1* and *NtARF10* were slightly upregulated in *MdCo41*-OE plants. This suggests that polar auxin transport exerts morphological changes in transgenic plants.

IPT (isopentenyl transferases) is a rate-limiting enzyme in CK biosynthesis. Overexpression of *AtIPT* accelerates plant branching by elevating endogenous CK levels [51]. A negative correlation between GA levels and axillary meristem formation in plants has been reported. In peas, gibberellin inhibits the growth and development of lateral branching. Silencing *AtGA20ox1* decreases stem and hypocotyl length by reducing endogenous GA levels in transgenic plants [52]. By contrast, the overexpression of *OsGA2ox* reduces tillering by specifically inactivating C_20_-GA precursors in rice [53]. Transcripts of *NtGA2ox* and *NtGA20ox* changed dramatically in *MdCo38*-OE but not in *MdCo41*-OE plants.

Carotenoid cleavage dioxygenase CCDs play an important role in carotenoid metabolism, especially in SLs biosynthesis. *CCD7* and *CCD8* regulate lateral shoot and root development in plants. *AtCCD7* and *AtCCD8* influence the growth of lateral buds and roots in Arabidopsis [54]. *MAX4* is a homolog of *CCD8* in planta. *CpMAX1a* in *Chimonanthus praecox* encodes the cytochrome P450 protein (Cyt P450), and its ectopic expression restores branching phenotypes of Arabidopsis *max1-3* mutants by upregulating *AtBRC1* [55]. In our present study, the SL-related genes *NtCCD7*, *NtCCD8*, and *NtMAX4* were highly upregulated in *MdCo41*-OE, but at a low level in *Md38*-OE plants, meaning that SLs are involved in leaf growth.

GRF plays vital roles in growth and developmental processes such as chloroplast proliferation and regulation of cell size [56]. The *atgrf1/2/3* triple mutant exhibits smaller and narrower leaves and shorter petioles; correspondingly, overexpression of *AtGRF1* and *AtGRF2* leads to leaf enlargement by regulating cell expansion [57]. Likewise, *AtGRF5* promotes cell division, inducing leaf enlargement in transgenic plants [58,59]. No significant expression of *NtGRF*s was observed in transgenic tobaccos.

## 5. Conclusions

In the present study, two important genes, *MdCo38* and *MdCo41*, were selected from 11 candidate genes at the *Co* locus. *MdCo38* is localized in the cell membrane, and *MdCo41* is localized in the nucleus, cytoplasm, and cell membrane. Overexpression of *MdCo38* and *MdCo41* resulted in branching and enlarged leaf size in transgenic tobaccos, respectively, by regulating auxin, GA, and SLs. Together with *MdCo31*, *MdCo38* and *MdCo41* are involved in the columnar growth of apples, which provides new insights into the genetic and molecular regulation of the columnar growth habit, and for further apple breeding through genetic transformation.

## Figures and Tables

**Table 1:** Isolation of predicted genes at the *Co* locus and expression analysis in apples.

Serial No. of Predicted Genes	Reported Genes	Genome No.	Putative Function	Gene Expression through SRA/EST Mining	Differential Expression in Shoot Tips of Columnar Apple	The Length of Genes in Genomic DNA and cDNA
*Co4*	*MdCo4* [18]	MDP0000897594	MYB Transcription factor 30- like	Fruit, root, shoot tip	1.6-fold down regulated	gDNA 1573 bp/cDNA 804 bp
*Co6*		MDP0000326311	Pre-mRNA-splicing factor SLU7	Fruit, leaf, root, shoot tip	2.8-fold down regulated	gDNA3204bp/cDNA1623bp
*Co9*	*MdCo9* [18]	MDP0000942873	Inorganic phosphate transporter1-4	Fruit, leaf, root, shoot tip	4.1-fold down regulated	gDNA 626bp/cDNA 626bp
*Co10*	*MdCo8* [18]	MDP0000329966	Actin depolymerization factor (ADF)	Fruit, leaf, root, shoot tip	2.1-fold down regulated	gDNA1516bp/cDNA450bp
*Co11*	*MdCo6* [18]	MDP0000284965	Root UV-B sensitive protein (RUS)	Fruit, leaf, root, shoot tip	1.6-fold down regulated	gDNA 3232bp/cDNA1503bp
*Co31*	*MdCo31/32* [18]	MDP0000687812	2OG-Fe(Ⅱ)oxygenase	Root, shoot tip	62-fold up regulated	gDNA1631bp/cDNA1020bp
*Co32*	*MdCo39* [18]	MDP0000912170	Walls are thin 1(WAT1)-related protein	Fruit, leaf, root, shoot tip	2-fold up regulated	gDNA1735bp/cDNA1050bp
*Co33*	*MdCo40* [18]	MDP0000912172	Unknown	Fruit, leaf, root, shoot tip	14-fold up regulated	gDNA 411 bp/cDNA 411 bp
*Co37*		MDP0000934869	A putative ADP-glucose phosphorylase	Fruit, leaf, root, shoot tip	2.9-fold down regulated	Gdna 432 bp/cDNA 432bp
*Co38*	*100016-gene* [18]	MDP0000163720	1-aminocyclopropane-1-carboxylate oxidase (ACO)—like	Fruit, leaf, root, shoot tip	4.7-fold up regulated	gDNA1437 bp/cDNA1083 bp
*Co41*		MDP0000684988	Pentatricopetide repeat (PPR)	Fruit, leaf, shoot tip	34.8-fold up regulated	gDNA 672 bp/cDNA 672 bp
*Co44*	*95062-gene* [18]	MDP0000878773	ACO-like	Fruit, leaf, root, shoot tip	2.3-fold down regulated	gDNA1304 bp/cDNA 1041 bp

## Data Availability

All the data are available from the authors upon a reasonable request.

## Supplementary Materials

The following supporting information can be downloaded at: https://www.mdpi.com/article/10.3390/genes14050964/s1, Table S1: Primers used in this study; Table S2: Sequence alignments of 11 candidate genes in ‘McIntosh’; Figure S1: Phylogenetic analysis of individual gene at the *Co* locus mentioned in the study; Figure S2: Phenotypes of transgenic tobacco by overexpressing *Co32* (A) and *Co33* (B), and analysis of several growth parameters (C~F); Figure S3: Expression analysis of *MdCo38* (A) and *MdCo41* (B) in apple cultivars and F1 population of ‘Jinlei No.1’ × ‘Maypole’. (XU, GL3, JL1, WL, and WSK represented ‘McIntosh’, ‘Gala 3’, ‘Jin-Lei No.1’, ‘Tuscan’, and ‘Wijcik’, respectively).
