# Supplementary material for: Screening Candidate Genes at the Co Locus Conferring to the Columnar Growth Habit in Apple (Malus × Domestica Borkh.)
Source: Genes (Basel). 2023 Apr 24;14(5):964. doi: 10.3390/genes14050964 (PMC10217585; doi:10.3390/genes14050964)
Supplement: Supplementary file 1 [file genes-14-00964-s001.zip › genes-2266984-supplementary.pdf]

Supplementary materials

Table S1. Primers used in this study

	Forward primer	Reverse Primer
MdCo4	TTAGTGCTCCTTGCTGCC	TCAAATTCCTAACAATTCTGGAATTTCC
MdCo6	CGACTGCTTCAGGGT	TTAGTTGAGAAAGTCCTTCATTGG
MdCo9	ATGAATGCTATTGAAGAAGTTTACAGAA	CTACACAACTGGAACTGTCCTGTTG
MdCo10	CTGCAATGTCTTTCAGAGGGC	TCAATGGGCACGGTCTCTGAGCA
MdCo11	ATGACGTCCTTTTCGCTTG	TCAATATCTTTGAGAATAATTATCAGTACC
MdCo26	AGACATTAGATCAGAATCTT	TTAGCTACTCGAGAGGCTTAAA
MdCo28	ATGATGTTCGGTAGGTTGAAGGGTG	CTATGGCTTTATTTCCGGAGTTTCTGG
MdCo32	ATGTTGCAGTTAATGAGCTGTGGC	CTACTCATTACTAGTTAATATAGTTTGCTCT
MdCo33	ATGAGGCAAAGAGAGATGCTGGAA	TCAACACCAACCTTGAGGCCAC
MdCo37	ATGGCATCAGATTCAGCAGCT	TAAACTTAATAAAATATTAAAATAAGTATTTAAAG
MdCo38	AAGACGAATCCTCGTATTACG	TTAGGCTAATTTAAAATGAGAAAGGGCA
MdCo41	ATGTCAACTGTTGTTTCGGCTTGTG	TTAAAAACTGCACCACCATAACTCAGAGTC
MdCo44	TGGACGTGTTGGATCA	CTAAAGTTTGTAACGTTTCATGTATTCTC
qNtGRF1	ATAAACAGAGGTCGCCATCG	GTTGACAAGCTGCTCAGTGG
qNtGRF3	CAGAGCCAGGGAGATGTAGG	CACCACTAGCATGGCCACTA
qNtGRF5	CTCTGCGAGGTTCTTCCATC	TGCTGCTTCCAGTAGCATTG
qRT-MdCo41	AGAAGAAGGCCGCAAGAGATT	GCTCGCAGCCACCTAACAAT
qNtMAX4	ATTTATTAAAGGCTGAGCCCACA	ATCCTCGCCGTTGAATGACC
qNtCCD7	TGCTTGTGGGAAGGTGGTGA	ATCCTCTGCGTTGCATGACG
qNtCCD8	GGCTTAGGTACTTCGGAAAA	AGAAATGGCCCAGGACAATG
qPCR-NtIPT	AACGCAAAGGTATCCCTCA	CATCAACCCAAATAAAGCAAC
qPCR-Co38	GTTCCTCATTCATCCACCAC	CCACTCCATCTTCACCTCC

qRT-GA20ox	CATAGGCGATACATTTATGGCG	CAGCTCTGTAATGCTTTTGTGT
qRT-NtGA2ox	GTGCTGGAAATGATAGCAGAAG	AAAAGGAGTAGTGATCAGGTGG
qRT-NtIPT	AACGCAAAGGTATCCCTCA	CATCAACCCAAATAAAGCAAC
qRT-NtPIN1	ATTAGGAACCCGAACACTTA	ACACCCATTGAGAAAGCAG
qRT-NtPIN2	TATTCTGTTCAATCGTCCCG	CCCGCTCATACTCCTACCTC
qRT-Ntactin	AGAGGTTCCGTTGCCCAGAAGT	GCTAGGAGCCAAAGCCGTGATT
qRT-MdACTIN	GGATTTGCTGGTGATGATGCT	AGTTGCTCACTATGCCGTGC

Table S2. Sequence alignments of 11 candidate genes in ‘McIntosh’

Genes name	‘McIntosh’ sequence
Co4	ATGGTTAGTGCTCCTTGCTGCCAGAAGATGGGGTTGAAGAAAGGGCCTTGGACTGCAGAAGAAG<br>ATCACATACTTGTCACTTACATTCAGCAGCATGGCCATGGAAACTGGAGAGCTCTCCCAAAACT<br>AGCTGGTTTGTTACGGTGTGGAAAGAGTTGTAGACTCAGATGGACAAACTACTTGAGGCCAGAT<br>ATCAAAAGAGGGAATTTTAGCAGGGAAGAAGAAGACGCCATCATCAACTTACATCAAATGTTA<br>GGAAATAGATGGTCACAAATCGCAGCGAGATTGCCTGGTCGCACAGACAATGAAATAAAAAAT<br>GTATGGCACACCCATTTAAAAAAAAAGCTGAAATCGAACCACACCGAACCAAATTCTAGGGCA<br>CGTTTCTACTCTAGAAAATCGGAGCAAGAGCTAGAGCCCATTGATGATTCTTCATATTGTGCAAA<br>ATCTGATAGCTTGGATTGTGTGCCTGTTTCGCCGGCTCGATGTACTTCAAATAACACGTCATCTGT<br>CACGACCAACGACTACGACAACAACAACAATAGTAACAATGTATGTGTGACATTTGACGATTAC<br>CAAGCAAATTTACCAGAACCGGATGATAGTTTTTGGTCGGAAGTTTTGTCAACTGAGTATTATGA<br>AGAGACGGTGAATGAGTTTCAGGCATTTGGTGGTGACCCACAAGTGAATATGGGGCCTACTGAT<br>GGTGGTTTTAGTTTAGACATGTACAATGACAACAACATGGACTTTTGGTTTAATATATTCTCAGG<br>AGCTGAGGAAATTCCAGAATTGTTAGGAATTTGA
Co6	ATGGCGACTGCTTCAGGGTCATTCAAATCTAGGGAAGACCATCGTAAGCAAATGGAATTGGAAG<br>AAGCGCGTAAAGCTGGGCTTGCGCCAGCTGAACTGGACGAGGATGGAAAGGAGATTAATCCTC<br>ATATTCCCCAATATATGTCATCTGCGCCTTGGTATCTCAAACACGAGAGGCCTAGCTTGAAACAT<br>CAAAGGAAATGGAAATCAGATCCAAATTACACAACATCATGGTATGACAGAGGTGCAAAGATA<br>TTCCAGGCTGATAAATATAGGAAGGGTGCATGTGAGAACTGTGGTGCCAGTACACACGACTCAA<br>AGTCATGCATGGATAGGCCCAGGAAAGTGGGAGCAAAGTGGACTAACACGCACATTGCTCCTG<br>ATGAAAAGATAGAGACTTTTGAGCTTGACTATGATGGAAAACGGGACCGCTGGAATGGATATGA<br>TGCAACGAGCTATGCTTTGGTAGTTGAAAGATATGAATCAAGAGATGAAGCTCGAAAGAAATTC<br>TTAAAGGAACAGCAACTAAAGAAGTTAGAGGAGAAAAATAATAACCCAAATGGTGAGGACAA<br>GGTTAGTGATGTGGATGAGGATGAGGATGAGGATGAGGATGATTTGAGGGTAGATGAAGCCAA<br>GGTTGATGAAAGCAAACAAATGGACTTCGCAAAGGTTGAGAAGCGTGTACGTACAACTGGTGG<br>TGGAAGCACAGGAACTGTCAGGAATTTGCGTATTCGTGAGGATACTGCAAAATATCTCTTAAAC<br>CTTGATGTCAACTCTGCACATTATGACCCCAAAACCCGATCCATGCGTGAGGACCCTCTTCCAGA<br>TGCTGATCCGAATGAGAAGTTTTACGGGGGTGATAATCGATATAGAAATAGTGGTCAAGCCATG<br>GAGTTCAAGGAGCTCAATATCCATGCTTGGGAAGCATTTGAGAAGGGACAAGATATCCACATGC<br>AAGCAGCGCCATCCCAAGCTGAGTTGCTGTATAAGAATTATCAGGTGATCAAGGAGAATTTGAA<br>GTCAAAAACAAAGGACACTGTTCTGGAGAAGTATGGCAATGCAGCTAATGAAGAAGAATTTCC<br>TAGGGAGCTTCTACTGGGACAAAGTGAAAGAGAAGTTGAATATGATCGTGCTGGGAGAATCATC<br>AAGGGCCAGGATATAGCACTTCCCAGAAGCAAGTATGAAGAAGACGTCTATGTCAATAACCAC<br>ACCACTGTATGGGGTTCGTGGTGGAAGGATCATCAATGGGGCTACAGGTGTTGTAAGCAGGTTA<br>CTCGTAATAGCTATTGCACAGGTGCTGCTGGAATTGAGGCTGCTGAGGCTGCAGCTGATCTTATG<br>AAGGCTAACATTGCTCGCAAAGAGGCCAATGGTGATATCCCTGCACCAGTGGAGGAGAAAAGA<br>CCCGCTACTTGGGGAACTGATATACCAGATGATTTGGTTTTAGATGAGAAATTACTTGCTGAAGC<br>TCTTAAAAAGGAGGATGGAAGAAAGAGAGAAGAGAGAGATGAGAGGAAGCGTAAATACAATG<br>TTAAATGGAATGATGATGTTACTGCAGAGGATATGGAGGCTTATAGGATGAAGAAAGTCCACCA<br>TGATGATCCAATGAAGGACTTTCTCAACTAA
Co9	ATGAATGCTATTGAAGAAGTTTACAGAATCGCAAGGGCGCAAACTCTTATTGCATTGTGCAGTA<br>CTGTCCCGGGCTACTGGTTTACGGTAGCTCTCATTGACAGGATTGGAAGATTTGCAATTCAGTTG<br>ATGGGATTCTTCTTCATGACAGTGTTCATGTTTGCACTGGCTATTCCCTACGAACATTGGACTCAC<br>AAGGACAACCGAATTGGGTTCGTAGTGATCTACTCATTGACCTTCTTTTTTGCGAACTTCGGTCCT<br>AATGCAACCACATTTGTTGTGCCGGCTGAGATTTTCCCAGCTAGGTTCCGGTCTACGTGTCATGG<br>AATCTCAGCTGCGTCCGGGAAGCTTGGCGCCATAGTTGGTGCATTCGGGTTCTTGTACTTGGCTC<br>AGAACAAAGATAAGAACAAGACAGATGCAGGGTACCCTCCAGGCATCGGGGTTAAAAACTCGC<br>TCCTTGTGTTGGGTGTGGTCAACTTCTTGGGGATATTGTTCACTTTCTTGGTGCCTGAATCGAATG<br>GGAGGTCGTTGGAGGAGATGTCGGGTGAGAACGAAGAAGAAAGCGAAACCGGGACAGTGGAG<br>TTGGAGCAATCAAGCTATAACAACAGGACAGTTCCAGTTGTGTAG
Co10	ATGGCTGCAATGTCTTTCAGAGGGCTCAGTCGGCCAAATGCTTCATGTGCCATGGGGGTTTCTGA<br>TGAGAGCAAGAACACTTTCATGGAGCTACAGAGAAAGAAGGTTCACCGCTATGTGATATTCAAG

Co11	GTTGAGGAGAAGAAGAGGGAGGTCGTAGTTGAAAAGATTGGTGGTCCGGCGGAGAGCTATGATGATTTTGTGGCAGCTTTGCCTGATAATGATTGTCGATATGCCGTATATGACTTTGATTTCGTAACTTCTGAGAACTGTCAAAAGAGCAAGATCTTCTTCATCGCATGGTCCCCTTCGACCTCTCGAATCCGTGCGAAGATGCTCTACGCCACATCTAAAGACAGGTTTAGGAGGGAGCTGGACGGTATCCACTACGAGATTCAGGCTACTGACCCAACAGAGATGGATCTTGAGGTGCTCAGAGACCGTGCCCATTGAATGACGTCCTTTTCGCTTGTGGGCCTCAAATTGTCATTACATAGCACGATGTCGTTTCACTTGTTCCTTGAATTTATTTCCGGGCGGTTTGGCCCTCCCTATTCTGCCGCCGGAATTCTTATCCCCTTTGTACAGTCACCCATTTCACTCCACCACCTTCCCCCTCCGATGAAATTGTTGGAGAAGATAATGATGCGGAAGAAGGAGCCAGATAAAGCTCCAGCCCCTCCTGAGGTTCCGGCGTACTGGATCGAAACCTCCGAGTCGGTCTCCTACCGCTGCGAGTTTGACCCCAGCGGTCAACTCTCTGTGAAGATTCTTGATGACTCGAGACCAGTGTTCCTTAAGGTCGCTGACTCGTTCGTCAATAAATTTTTCCCCTCGGGATATCCATATAGTGTAAATGAAGGATATCTCAGATACACCCAATTCCGGGCACTGCAGCACTTAACCAGTGCAGCCCTGTCAGTGCTTTCAACTCAGTCACTCCTATTTGCTGCAGGCTTGCGACCAACCCCTGCACAAGCAACTGTTGTAAGTTGGGTTCTAAAGGATGGGATGCAGCACATGGGAAAGCTTATATGTAGTAATCTGGGCGCAAGAATGGATTCCGAGCCTAAATGTTGGAGAATTTTGGCTGATGTGCTCTATGACTTTGGCACTGGCTTGGAAGTTCTTTCTCCTTTGTGTCCACAGCTTTTTCTTCAAGTTGCAGGTTTTGGCAATTTTGCAAAGGGGATGGCAGTTGTTGCAGCGAGAGCAACAAGATTACCAATTTATTCAGCCTTTGCAAAAGAGGGCAATCTTAGTGATCTGTTTGCCAAAGGGGAGGCAATCTCAACTGTGTTCAATGTCATTGGAATGGGAGCAGGGATCCAATTAGCATCTACTGTCTGTTCGTCTATGCAAGGAAAGATGGTTGTTGGGCCTCTTCTTTCAATGGTACATGTATACTGTGTCATTGAAGAAATGCGGGCAACTCCTATCAACACGTTGAATCCACAGAGGACTGCAATGATTGTGGCTGATTTTCTGAAGACGGGAAAAGTATCAAGCCCTGCTGACCTGAGGTATCGGGAAGACCTCCTATTCCCTGGGCGACTCATAAAGGATGCCGGAAATGTGAGAGTGGGAAGGTCATTCCACGAGGTCATTAAGCCTTCAAAACTTGGTGAATTGAAAGGAATGTTCCCTGAAGAGAAGTTTCTTTTAAGTCGTGGAAACAAAGGGGTTGACATGTTATTGGAGAAAAATGCTACCGGCGAAGACGCATTGAGGGGGTGGCTTGTTGCTGGATACACTGCGGATATAGAGAAGTCTTTTCGTGTGCCGAGTCCGAGCGCACTGGAAGAGGCTTACGAGAAGATGAATGACGTATTTGGTCCATTTGTATTGGAACTGCAGGCCAAGGGGTGGCATACCGATCGATTTCTCGATGGAACAGGAAGCCGTTTTGCGTGGGGGTCGAATCTAGGTACTGATAATTATTCTCAAAGATATTGA
Co32	ATGTTGCAGTTAATGAGCTGTGGCACCTGTTACAGGATGGGCTTCGCAAGATTCAAGCCGCACATTCTCATGGTTTTGGCACAGATCGGTTATACATTTCTCTACTTGATTACAGCTGCATCCTTCGATCATGGGATGAACCCTCATGTCTTTGTAACCTATCGACATATAGTAGGTGGCTTAGTGATGTTTCCATTTGCCTATTTTCTTGAAAGAAAAGTAAGGCCAAAGTTGACACTAGCAGTGTTTCTGGAGATATTTTTACTTTCTCTGCTAGGGGTTAGCTTAACCCTTAATTTGTATTTTGCGAGCTTGAAGTACACGTCTCCAACCTTTGTCACATCGGTCGCCAACACCATTCCGTCCATAACTTTTATAATTGCAGTCATACTCAGGATGGAGGCTGTAGATGTTAGGAATCCCCGTGGAATAGCTAAAATCTTCGGAACCTTAATATCTTTGGCTGGAGCCATGACCATGACCTTGTACAAAGGACCTGCCGTGCGAAGCTCACCAGGAGCTCCAAAACACATCACAAGTAACTCTGTTCATGAAAACTGGACGAAGGGATCAATCCTCCTCATTGGAAGTTGTATATCATGGTCCGCATGGTTCATAATGCAGGCAATCACACTGAAGAAATATCCTGCACAACTGTCACTAACCACATGGATAAATTGTATTGGGGCAGCACAATCGGCTGTTTTCACAGTAACAATACAACACAAAAGGGTAGCATGGAGTATTAGATACAACAATGACTTCTGGTCCATCATCTATGCTGGAGTTGTATGCTCCGGTATAATGGTCTTCATTCAACTGTGGTGCACAAAACAAAAAGGACCAGTTTTTGTGACCATGTTCAGTCCCCTTGCAACGGTATTGGTGGCAGTTATGGCATACTTCATTCTTGGTGAAAGACTGCACGTTGGCAGAATACTGGGAGCAGTCATTATCACCATCGGTCTCTACATGGTATTATGGGGCAAAGATAGAGATCAAAATGTTATCAGGTCGCAAGAGCAAACTATATTAACTAGTAATGAGTAG
Co33	ATGAGGCAAAGAGAGATGCTGGAATTTAGGACACCCCAACACCCATCGTCAATGGGTTCGGGGACAAACACTTGGGGTGGTGCTTCTCCTTTGTTGGCAAGAAATATACCCAAAGAGTCACTTGAACAGAAGTATCTCAGGCTCAACACGATACGAACGCGCGATGAGATCTTCCCGGCTAAGGATGATGAGTTTGACAACCTACCTAAGACTCCTCCGCATAAACAAGAGTGTCCTCCCACGCCGAGCAGAAGCAGAAAACAATATATAATGAAGCTTTTGTTCACACCAAAGAGGAATAAGAGAACTACTACGAGGACGATGGATACTGCTACACTGGGTTGGAATTCGAAGAAGTCGTGGTTTCCTCGAATGGATCCGAAAAAGAGGTGGCCTCAAGGTTGGTGTTGA

Co37	ATGGCATCAGATTCAGCAGCTCGAACCCCGCAACTGAGGAAAGACTCGGTGACGAAGCGGTGG<br>GTGATATTCTCACCCGCCAGAGCCAGGCGACCTTCCAACTTCAAATCCAAGTCCCCCATCAACC<br>CCAACTCCGACCAACAACAACAGTGCCCCTTCTGCATCAGCCACGAGCACGAGTGCGCTCCCGA<br>AATCTTCCGGCTCCTGCCCGATAACGCCGACTGGACAATCCGGGTCATCCAGAACCTCTACCCC<br>GCCCTCAGCAGAGACATGGAAGTCGTCTCTGCTCAAGACCCGTTACACCAGAACTCCGATTGGG<br>TGATGGGGGATGGGGGAAGGCGTGGGAGGGTGGGCACGGGTGGGGATCTGTGTTTTTTTTTTTTT<br>TTTTACTTTTCTTTAAATACTTATTTTAATATTTTATTAAGTTTATAG
Co38	ATGGAAGACGAATCCTCGTATTACGACAGAACCAAGGAAGTGAAAGAGTTTGATGAAACGAAA<br>GCCGGAGTTAAGGGCCTTGTGGACTCAGGGATCACAAAAGTCCCCAAGTTCCTCATTCATCCAC<br>CACATAGCCTTTCAAATTCAGAAACAACAAATGTAGACTTCAAGGTTCCTGTCATAGACTTTTAC<br>GGCTTTGATCAAGATTTCCGGCGAGCGCAGATTGTGAAGGAGATTTGTGAAGCATCCGAAACGT<br>GGGGGTTCTTTCAAATGGTTAATCATGGAGTTCCAGAGAGAGTGATTGAGAATATGTTGGAAGG<br>GATTCGAGGGTTTCATGAGCAGCCGAAGGAGGTGAAGATGGAGTGGTATTCTCGTGACTCGAAA<br>CATAGAGTGAGATACTACTGCAATGGAGATCTGTTGGTGTCGAAAACAGCGAATTGGAGAGAC<br>ACGATAGGGTTTGATTTCCAGGACGGTCCATTGGACCCCGAGACATTTCCCTTCGTTTGCAGAGA<br>GGCAGTGCAGGAGTACATCGATCACTTAAAGAACTTGAGGGAGATGCTATCAGGTTTGTTGTCC<br>GAGGCCTTGGGGCTCAGCACTGACTACCTGGAAAACATAGAGTGCATGGAGACTGAAAATCTG<br>GTGTGCCATTACTACCCGAGCTGTCCAGAACCGGAGCTGACCCTTGGCACAACCAAGCATTCGG<br>ATCCTTCCTCTTTAACTGTACTCTTGCAGGATAATCTCGGCGGCCTGCAAGTCCTCCATCAAGGTC<br>ACTGGGTTGATGTTCCTCCAACTCCTGGAGCTCTTGTGGCGAATGTCGGTGATCTTATGCAACTTA<br>TCACCAATGACAAGTTCAGAAGCGTCGAGCATAGAGTACTGGCTAGAAGAGTGGGGCCCAGGA<br>TCTCAGCTGCATGTTTTTTCTATCCAAGTGCTACGCAAAGATTCAAACCCTATGGCCCCATCAAG<br>GAGTTTCTATCGGACAACCTACCGATATACAGGGAAACACACTTTGGGGAGTATCTTGCTTATTA<br>CAGATCAAAGGGGTTAGATGGTAATTCTGCCCTTTCTCATTTTAAATTAGCCTAA
Co41	ATGGAAGTGCTCGGAGTTCACGGATATGCAAATGAAGCAACAGCAATGGTCTGCAAGATGGAG<br>AGAGAGAAGATCAAGCCAAATGGGGTTACTTTTGAAAGTGTTTTAAGTGCCTGCACTCATTCAG<br>GATTTGTAGAAGAAGGCCGCAAGAGATTTTCAAGCACGATCCTGGACTATTCCATCCGCCCCAA<br>AGTTGAGCAATATGGATGCATGGTGGATCTGTTGAGCAAAGCCGGCCTGCTTGAAAATGCTCTA<br>GGGTTGATAAGAAGCATGGAGTTTGAACCGAACTCTGGTATATGGGGTGCATTGTTAGGTGGCT<br>GCGAGCTTCGTAAAAACTTGAAGATTGCTCAAGTTTGTGTCAAGGAATTGATGTTGTTGGAGCCA<br>AATAATTGTGGGTGTTTCAACCTTTTGGTGAACATGTGTGTGGATGCAAAACGATGGGGAGAAGT<br>TGCGGATATTCGAGCAACCATGAAGGAACTTGGAGTTGAAAAGGGATGTCCTGTGTCCAGTTGG<br>ATTGAAATGGAGAGGAAAGTTCATCAGCTTGCAGCGTCTGATGAATCTCATTCAGCTTCTGATGT<br>AATTTACTCATTGCTGGTGGAATTATATGTGCAGCTGAAACTTGACGTTTATGTTCCTGAACTTGA<br>CTCTGAGTTATGGTGGTGCAGTTTTTAA
Co44	ATGGTGGACGTGTTGGATCATGAGCTGGAGGAGGAACTGAAACAGTTCGACGAAACCAAAGCT<br>GGTGTGAAAGGGCTTGTAGATTCTGGGGTGACCAAGCTCCCAAGAATGTTCAAACACCCTCCGG<br>AGTGTCTACCATCCCCGAACCAAAACAATGGAATTCACGACCTGCAAGTTCCTGTCATTGATCTG<br>AAAGATGCGGAGAACAATGAAAGGAGAAGCGAAATCATCAGTAACCTCCGCAAAGCAGCTGA<br>AGAATGGGGTTTCTTTCAAATTGTGAATCATCCCATTCCACTTGACGTCATGGATGATGTGCTGA<br>AAGGTGTTCGGCGGTTTCATGAGCAGCCCCAGGAAGCCAAGGAGGAGTGGTATTCGCGTGATTT<br>CACGAAGAAGGTCAACTTCTTCAGCAATGGAGAATTGAAGGTAGACACTCCAGCTGACTGGAG<br>AGACACCTTGTCGTGCAAAGTCCTCGAAGATGAACGAAACTTTGAAGAAATCCCTGAAGTCTGC<br>AGAACGGAAATACGTGAGTATATGAAATACATCGTTCAAGTGAAGGAGAAGCTATCCGGATTAT<br>TTTCGGAAGCTTTAGGCCTAAGCAGGGATTACCTTGAAAATCTAAGGTGCTTCAAATCTAGGTCA<br>TTGGCATGCCACTACTACCCGGTATGCCCGGAGCCCCATTTAACCCTAGGAGGAACCAAACACT<br>CCGATCTCGGCTTTCTAACCTTGCTCTTGCAAGATAGTGCTGGCCTACAAGTTCTTCATCAAAATA<br>TTTGGATTGATGTTCCCCCAGTGGAGGGAGCTTTGCTCATCAATCTTGCCGACATGCTGCAGTTTA<br>TTACTAATGGCAAGTTCAAGAGTGTACAGCACAGAGTACTGATGCCACTGTCGACCCTCGAGCC<br>CCGTACGTCAATTGCATGTTTTGTTGGCACGGACGACCTCCAAAAACCTTACGGTCCGTTAAAGG<br>AGCTCGTCTCCAAAAACAATCCGGCAAAATACGAAGATGTCTGTTTTGGAGAATACATGAAACG<br>TTACAAACTTTAG

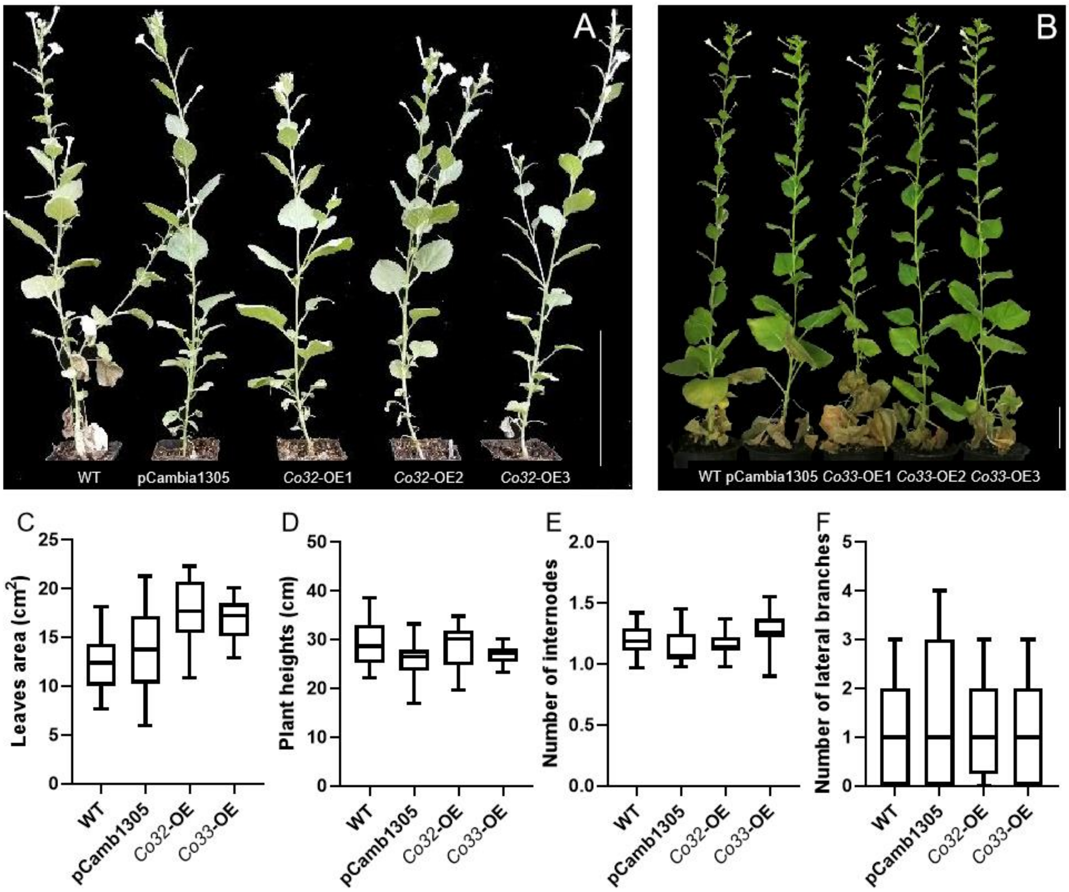

Figure S1 Phenotypes of transgenic tobacco by overexpressing *Co32* (A) and *Co33* (B), and analysis of several growth parameters (C~F)

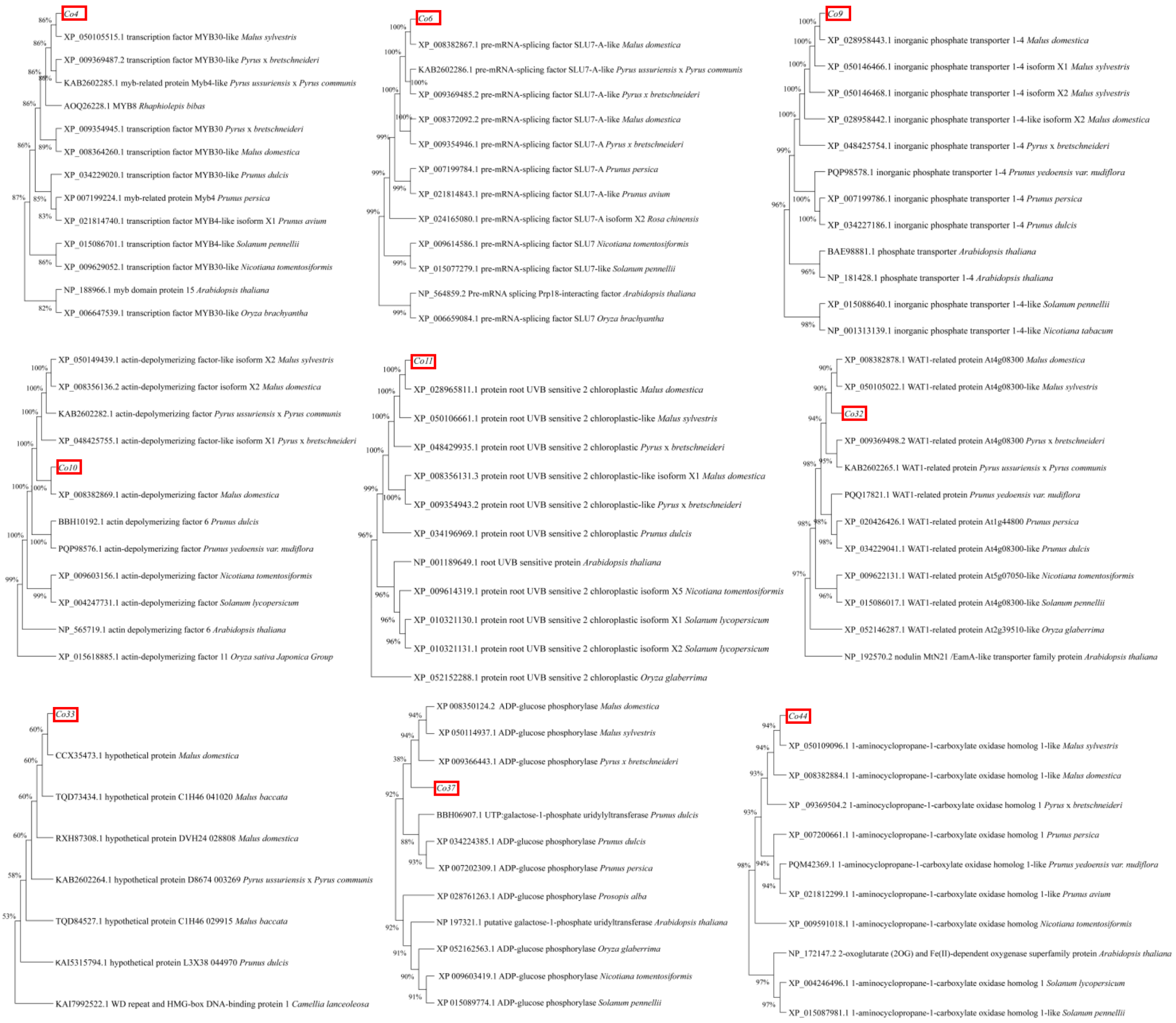

Figure S2 Phylogenetic analysis of individual gene at the *Co* locus mentioned in study

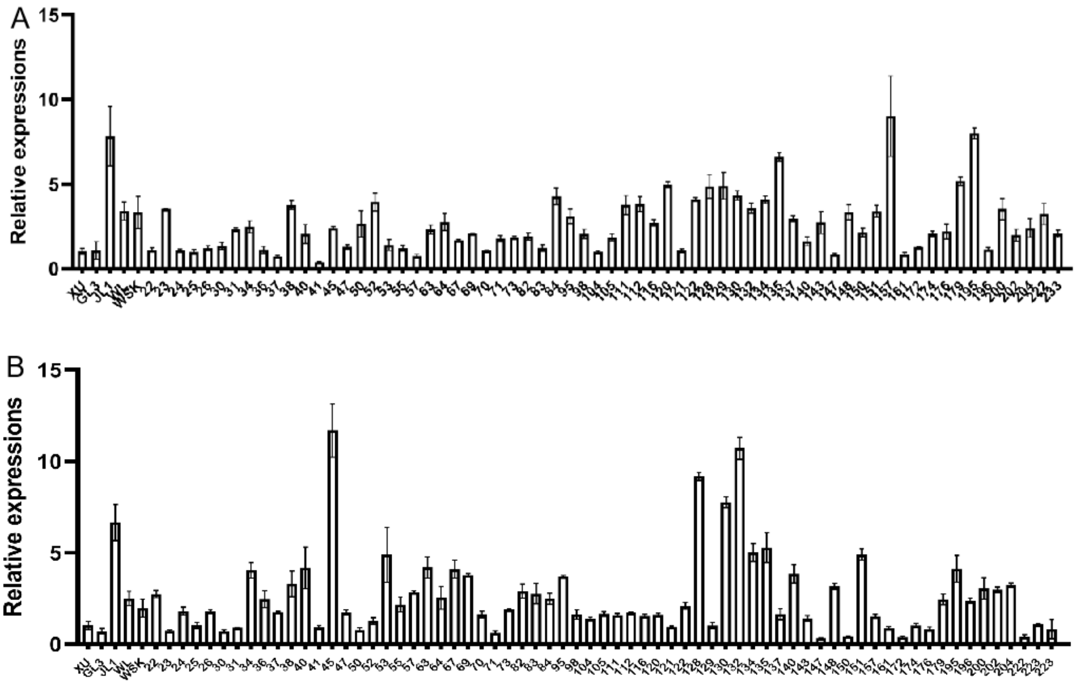

Figure S3 Expression analysis of *MdCo38* (A) and *MdCo41* (B) in apple cultivars and F1 population of ‘Jinlei No.1’ × ‘Maypole’. (XU, GL3, JL1, WL, and WSK represented ‘McIntosh’, ‘Gala 3’, ‘Jin-Lei No.1’, ‘Tuscan’, and ‘Wijcik’, respectively)
